# Research on the Performance Laws of the Piezoelectric Beam and Magnetic Coupling Module of the Magnetic-Coupled Double-Wing Negative Stiffness Energy Harvester

**DOI:** 10.3390/ma18071503

**Published:** 2025-03-27

**Authors:** Jie Yang, Yingchun Chen

**Affiliations:** College of Architecture and Civil Engineering, Beijing University of Technology, Beijing 100124, China; yj1998040221@163.com

**Keywords:** modal characteristics, output characteristics, vibration energy conversion, magnetic coupling

## Abstract

With the rapid development of urban rail transit, the floating slab vibration isolation system has become widely used in the field due to its effective vibration reduction and isolation capabilities. Traditional floating slab vibration-isolation systems mainly focus on blocking vibration transmission, neglecting energy harvesting. This paper proposes a magnetic-coupled double-wing negative stiffness energy harvester for floating slabs. A single-wing piezoelectric beam model and a finite element model of the magnetic-coupled module are established. The modal and output characteristics of the single-wing piezoelectric beam are analyzed. Furthermore, the force characteristics of the magnetically coupled negative stiffness module are analyzed. The results show that the contribution of its width to the modal frequency gradually decreases with an increase in the length of the single-wing piezoelectric beam. The thickness significantly influences the characteristic frequency, and the load is exponentially related to the output power. At the optimal load and characteristic frequency of the single-wing piezoelectric beam, the output characteristics decrease with an increase in the width. The peak value of the magnetic-coupled negative stiffness gradually decreases with an increase in the magnetic gap. The increase in remanent magnetic strength indicates that the initial state of the magnetic ring is more easily affected by external conditions. The change in axial magnetic force becomes significant with increased displacement. This research enriches the theoretical systems of piezoelectric energy harvesting technology and magnetic-coupled negative stiffness mechanism while providing important theoretical support for subsequent experimental research, optimal design, and practical applications.

## 1. Introduction

With the rapid development of urban rail transit, problems surrounding the vibration and noise caused by urban rail transit operations have gradually begun to attract the attention of researchers [1,2,3]. These problems seriously affect the riding comfort of passengers. They will likely seriously impact buildings and residents, causing irreversible damage to the precision testing and monitoring equipment around the tracks and platforms [4]. The floating slab vibration isolation system is widely used in light rail systems due to its effective vibration reduction and isolation capabilities [5,6]. Traditional floating slab vibration isolation systems mainly focus on the effect of blocking vibration transmission, almost neglecting the collection of vibration energy.

The design of the vibration pickup structure of the piezoelectric energy harvester is important, where the cantilever beam with a piezoelectric layer plays the role of a basic energy conversion structure [7,8,9,10]. Researchers have proposed a typical cantilever-type piezoelectric energy harvester to improve its performance [11]. This device comprises a cantilevered elastic beam covered with piezoelectric materials and a pointing mass [12,13]. The cantilever structure generates electricity through the *d*_31_ effect. The *d*_31_ effect refers to the phenomenon in piezoelectric materials where electric charges are generated in a direction perpendicular to the direction of mechanical stress when applied along a specific direction [14]. The cantilever elastic beam will undergo bending deformation when an external vibration is applied to the device, generating stress in the piezoelectric materials [15,16]. This stress will generate electric charges in the direction perpendicular to the stress, converting mechanical energy into electrical energy. Scientists have demonstrated that the natural frequency of the device can be adjusted through a rational design process that considers the dimensions of the cantilevered elastic beam, its material properties, and tip mass. This adjustment enables the device to align with the frequency of the external vibration source, enhancing the energy harvesting efficiency [15]. The selection and treatment of piezoelectric materials will also have an important impact on the performance of the energy harvester [16,17,18].

Alghisi et al. [19] proposed a piezoelectric energy-harvesting transducer consisting of a rigid ball. When externally excited, the ball repeatedly bounces between piezoelectric membranes arranged in a three-dimensional geometry and strikes the piezoelectric membranes, generating energy. Zhao et al. [20] proposed an electromechanical modeling solution. The authors experimentally validated collecting electrical energy from excitation under axial compressive loads in the non-resonant low-frequency range of a multi-layer piezoelectric stack. Li et al. [21] designed a piezoelectric cantilever beam collector based on polyvinylidene fluoride film. This collector comprises two cantilever mass systems to achieve two distinct resonant frequencies. The authors established a mechanical model theory to support this objective. Cao et al. [22] investigated the vibration characteristics of a cantilevered L-shaped beam oriented perpendicularly to the horizontal plane. The authors constructed a dynamic model using the Euler–Bernoulli beam theory. They derived a discrete model based on modal orthogonality. Liu et al. [23] proposed a quasi-zero stiffness (QZS) device for vibration isolation and energy harvesting. This device converts part of the vibration energy into electrical energy, reducing the energy transmitted to the receiver. Yu et al. [24] proposed a seesaw-shaped piezoelectric energy harvester capable of rotational motion and with the potential to enhance harvesting performance under low-frequency vibration. Initially, the authors completed the design of a harvester with a specific structure. They employed finite element analysis to simulate its dynamic characteristics.

Feng et al. [25] employed a high-impact accelerometer and a second-order oscillatory system to establish a dynamic response model of a bilayer heterogeneous structure and quantify its buffering and energy absorption capabilities. Song et al. [26] explored the conversion of low-frequency vibration energy into electrical energy. The authors observed the output frequency change with temperature. The structural design was inspired by the wing and improved performance through parameter coupling. Xu et al. [27] developed a piezoelectric energy collector to harvest vibration and wind energy simultaneously. Furthermore, the authors demonstrated the feasibility of powering low-power devices through experiments. Zhao et al. [28] established a coupling model of a vehicle-track vibration energy harvester. The authors designed the energy harvester with a new piezoelectric stack-type energy harvester with a double-arched frame structure. A relatively small amount of research has been conducted on the energy harvesting capacity of the floating slab vibration isolation system. Since this widely used system operates at frequent working frequencies with a large amount of energy that can be recovered and harvested, developing an energy harvester suitable for the floating slab vibration isolation system is imperative.

In this paper, a magnetic-coupled double-wing negative stiffness energy harvester with the capacity to harvest energy from the low-frequency vibration of the floating slab vibration isolation system is proposed. A common rectangular beam is selected as the piezoelectric base beam in the energy harvester. A simulation model and an experimental platform for a single-wing piezoelectric beam are established to explore the modal and output characteristics of the piezoelectric beam. Furthermore, theoretical and simulation models for the magnetic coupling module are developed to investigate the performance laws of the magnetic coupling module. The research contributes to the theoretical framework and performance law research of piezoelectric energy harvesting. Lastly, a theoretical basis for subsequent experimental research, optimization design of energy harvesters, and practical applications is provided.

## 2. Device Design and Static Analysis

### 2.1. Structural Configuration

**Figure 1:**
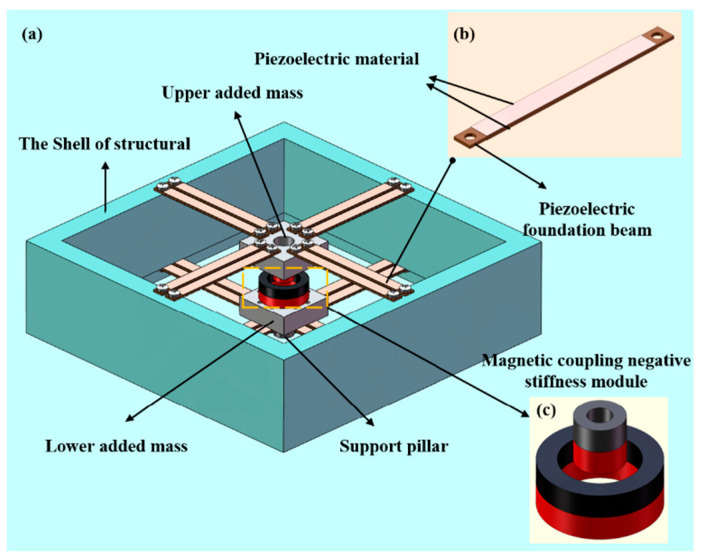
Structural schematic diagram of the magnetic-coupled double-wing negative stiffness energy harvester. (**a**) The magnetic-coupled double-wing negative stiffness energy harvester; (**b**) piezoelectric foundation beam; (**c**) magnetic coupling negative stiffness module.

The structure of the magnetically coupled negative stiffness module is shown in Figure 1c. The rectangular piezoelectric beam can maintain stable performance output throughout long-term use and is not easily affected by the external environment. The rectangular piezoelectric beam can maintain high-quality consistency during manufacturing due to its compact structure and ease of processing and manufacturing, improving its reliability [15]. A piece of PZT-5H piezoelectric sheet is arranged on each of the upper and lower surfaces of the piezoelectric beam, and a symmetrical arrangement is adopted to utilize the strain energy of the beam. The piezoelectric plates are connected in parallel configuration within the circuit of a single-wing piezoelectric beam. The lower magnetic ring is fixed to the upper surface of the lower additional mass, the upper magnetic ring is fixed to the lower surface of the upper additional mass, and the two axes are kept in a straight line and parallel to the z-axis. When no external load is acting on the structure, the magnetic coupling module is in the static equilibrium position, and the load is supported only by the piezoelectric beam. The upper magnetic ring will deviate from the equilibrium position if an external load is applied to the structure in the vertical direction. The mutually repelling magnetic rings can generate negative stiffness, used to offset the positive stiffness of the piezoelectric beam. Consequently, the displacement at the end of the additional mass is amplified at the natural frequency.

### 2.2. Simulation Parameter Setting

The piezoelectric base beam is made of elastic steel and beryllium bronze. In contrast, the material of the piezoelectric element is PZT-5H (lead zirconate titanate piezoelectric ceramic), which is the ideal material. Moreover, the influence of the size of the single-wing piezoelectric beam on the characteristic frequency and output performance is considered. The overall parameter scheme shown in Table 1 is adopted. In the finite element analysis, the extremely refined mode is used for the physical quantity-controlled mesh to divide the simulation model into grids, ensuring the reliability of the simulation results.

### 2.3. Experimental Setting

**Figure 2:**
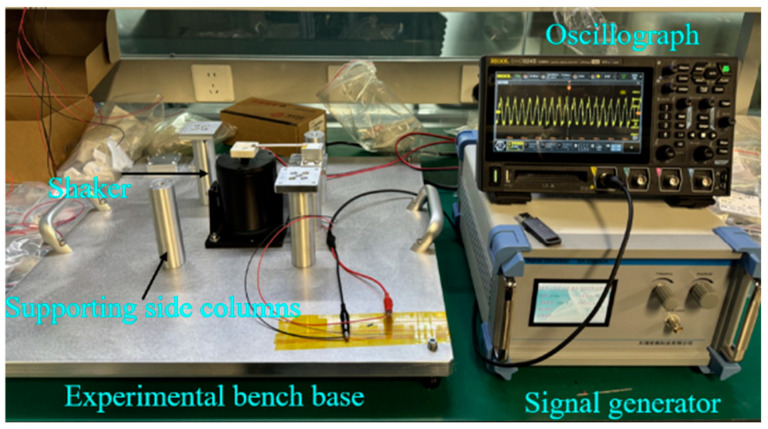
Magnetic-coupled double-wing negative-stiffness energy harvester testing system.

The output characteristics of the single-wing piezoelectric beam in the magnetic-coupled double-wing negative-stiffness energy harvester are experimentally investigated. The experimental setup, depicted in Figure 2, comprises an experimental bench base, a shaker, an oscillograph, and a signal generator. The signal generator generates and amplifies electrical signals, driving the shaker and producing vibrations. The vibrations drive the single-wing piezoelectric beam to vibrate and generate electrical signals. The signals are then collected, displayed, and stored by the oscillograph. The experimental parameters are shown in Table 2.

## 3. Results and Discussion

### 3.1. Single-Wing Piezoelectric Beam Performance

#### 3.1.1. Modal Analysis

**Figure 3:**
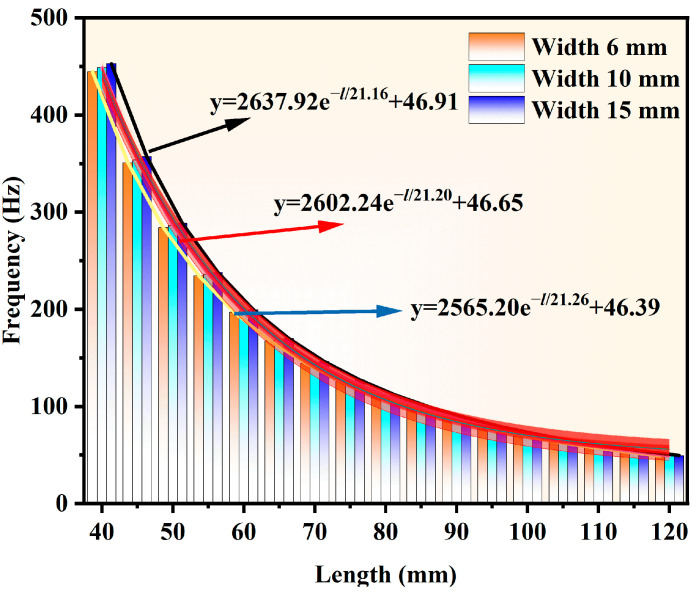
Fitting diagram of the influence of the single-wing piezoelectric beam length on the first characteristic frequency.

Figure 3 depicts the fitting diagram of the influence of single-wing piezoelectric beam length on the first characteristic frequency. The first characteristic frequencies of different widths of single-wing piezoelectric beams decreased exponentially with an increase in the length of single-wing piezoelectric beams. When the length increased by 200%, the first-order eigenfrequencies of 6 mm, 10 mm, and 15 mm-wide piezoelectric beams decreased by 88.97%, 89.03%, and 89.08%, respectively. The stiffness of the piezoelectric beam was weakened with an increase in length, decreasing the ability to resist deformation [27]. The beam was more likely to vibrate under the same external excitation, i.e., the characteristic frequency decreased.

Due to the nature of exponential decay, this frequency change was not linear. Instead, as the length increased, the frequency decreased and gradually accelerated. As the length increased, the characteristic frequencies of single-wing piezoelectric beams of different widths gradually approached each other. When the length of the piezoelectric beam was 60 mm, the differences in the first characteristic frequencies between the single-wing piezoelectric beam of 6 mm width and the other two single-wing piezoelectric beams of different widths were 1.31 Hz and 2.77 Hz. Thus, the effect of width on the first characteristic frequency of the single-wing piezoelectric beam was relatively small.

(1) y=2565.20e−l21.26+46.39

(2) y=2602.24e−l21.26+46.65

(3) y=2637.92e−l21.16+46.91

According to Table 3, when the piezoelectric beam was in the first mode, it mainly exhibited vertical vibration. Hence, the displacement and deformation along the beam width direction could be neglected [29]. In the initial mode, the piezoelectric beam’s output signal mainly came from vertical vibration. At the second to fourth characteristic frequencies, the piezoelectric beam started to experience varying degrees of displacement or lateral movement along the beam length as the frequency increased. Depending on the frequency, such displacement or lateral movement induced variable deformation in the piezoelectric beam. Additionally, this deformation could compromise the integrity of the piezoelectric materials at the beam’s upper and lower extremities. It should be noted that the piezoelectric material may exceed its tolerance limit if subjected to excessive deformation, which could result in material failure or performance degradation.

**Figure 4:**
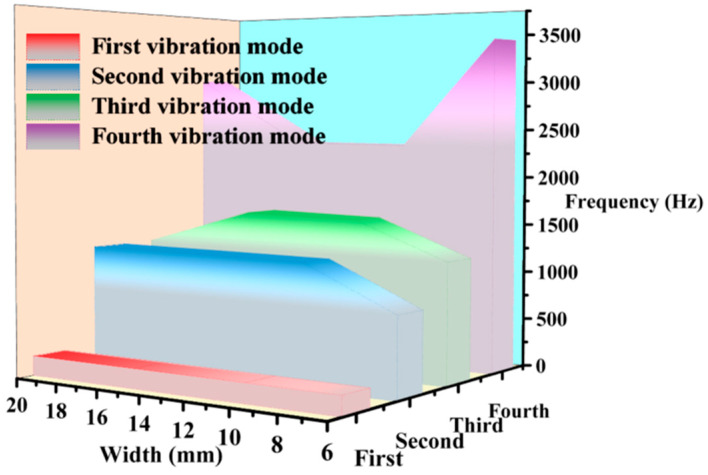
Fourth-order characteristic frequencies of single-wing piezoelectric beams with different widths.

Figure 4 represents fourth-order characteristic frequencies of single-wing piezoelectric beams with different widths. According to Figure 4, when the width increased by 14 mm, the first characteristic vibration frequency increased by only 2.21%, indicating that the width had a minor effect on the first characteristic frequency. When the piezoelectric beam was in the first mode, its vibration was mainly concentrated in the longitudinal direction. The first characteristic frequencies were 196.95 Hz, 198.25 Hz, 199.72 Hz, and 200.98 Hz for widths of 6 mm, 10 mm, 15 mm, and 20 mm, respectively. Changes in the width direction had a relatively small effect on the overall vibration characteristics.

The effect of the width on the fourth-order characteristic frequency of the single-wing piezoelectric beam was different. As the width increased, the second to fourth-order characteristic frequencies showed two stages. In the first stage, the secondary resonant frequency exhibited an upward trend with an increase in width. The frequency increased by 429.14 Hz from a width of 6 mm to 10 mm. The characteristic frequency demonstrated stability within a comparatively extensive range in the subsequent stage. As the width increased, the frequency increased by less than 7 Hz. This pattern of first increasing, then stabilizing, and finally decreasing reflects the complex changes in the piezoelectric beam’s internal stress distribution and vibration modes at different widths.

As with the second characteristic frequency, the third characteristic frequency demonstrated a propensity to increase, stabilize, and ultimately decrease. The increases in the third characteristic frequency were 367.1 Hz, 23.5 Hz, and −365.3 Hz for widths of 6 mm to 10 mm, 10 mm to 15 mm, and 15 mm to 20 mm, respectively. However, during the stable phase, the range of variation of the third characteristic frequency was smoother than that of the second characteristic frequency. This finding suggests that the vibration in the third mode exhibited minimal sensitivity to alterations in width within a specific width range. An augmentation in width significantly decreased the third characteristic frequency. This finding suggests that the impact of width on higher-order modes cannot be disregarded. Contrary to the trend exhibited by the third characteristic frequency, the fourth characteristic frequency initially decreased and subsequently increased. Specifically, the fourth characteristic frequency first decreased by 32.15% and then increased by 8.7% and 29.25% for widths of 6 mm, 10 mm, 15 mm, and 20 mm. This contradictory trend may indicate substantial variations in the vibration characteristics of the piezoelectric beam under different modes.

According to Table 4, as the width of the piezoelectric beam increases, the first characteristic frequency tends towards stability. The vibration of the piezoelectric beam in the first mode is primarily influenced by its length and overall structure. The observed change in the width of the beam exhibited a comparatively negligible direct effect on the first characteristic frequency [30]. The influence of the beam width gradually became more significant with an increase in the modal order. For the second to fourth characteristic frequencies, the piezoelectric beam experienced varying degrees of displacement or lateral movement along the direction of the beam length. This phenomenon caused the beam to undergo torsional bending. This phenomenon is characterized by the torsional bending of the piezo beam and by inducing damage to the piezo materials located at the superior and inferior extremities of the base beam. The internal stress distribution will be uneven if the piezoelectric material is subjected to excessive deformation or torsion. Consequently, material failure or performance degradation may occur [31].

The higher-order modes of piezoelectric beams were subjected to irregular torsion or bending, and the risk of damage to the piezoelectric material was increased. At the first-order characteristic frequency, it was more challenging for a narrower piezoelectric beam to demonstrate complex torsional and bending behaviors during vibration. However, as the beam width increased, the vibrational behavior of the beam gradually became more stable in higher-order modes. A wider beam exhibited greater inertia and stability during vibration. More attention was allocated to the structure’s first- and second-order modes to achieve optimal vibration control and energy conversion effects.

**Figure 5:**
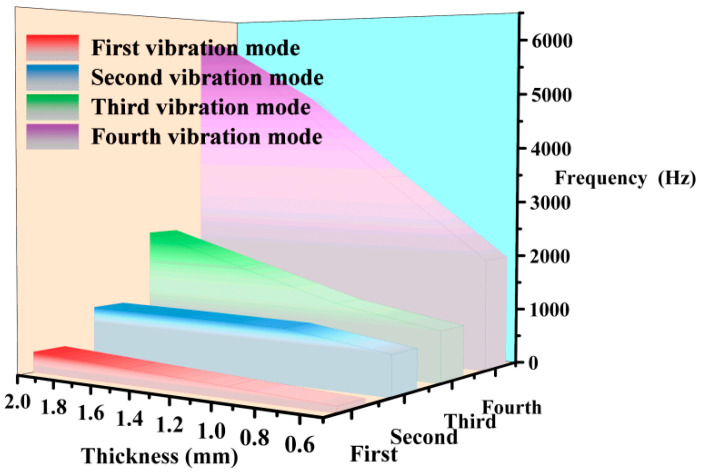
Fourth-order characteristic frequencies of single-wing piezoelectric beams with different thicknesses.

As demonstrated in Figure 5, an increase in thickness significantly enhanced the first characteristic frequency of the single-wing piezoelectric beam [32]. When the thickness of the single-wing piezoelectric beam was increased by 300%, the four characteristic frequencies increased by 222.57%, 40.40%, 150.96%, and 198.39%, respectively. Thickness significantly affected eigenfrequencies, and the sensitivity of the characteristic frequency of different orders to changes in thickness varied. Even though the second characteristic frequency was increased, it was relatively modest, with a percentage rise of only 40.40%. This finding suggests that the influence of thickness on the second eigenfrequency was less pronounced than that on the eigenfrequencies of other orders.

As demonstrated in Table 5, an increase in the thickness of the piezoelectric beam consistently increased its first characteristic frequency. However, the second characteristic frequency exhibited a sequence of behaviors, initially undergoing torsion and subsequently experiencing lateral displacement. Furthermore, as the thickness of the piezoelectric beam was augmented, the vibration mode of the beam at the second characteristic frequency underwent a sequence of events, initially twisting and then becoming displaced laterally.

The number of orders in which the single-wing piezoelectric beam with a thickness of 0.5 mm underwent irregular torsion was higher than that of the 1 mm and 1.5 mm single-wing piezoelectric beams. The complex vibration mode caused the piezoelectric beam to generate excessive stress and deformation during vibration, indirectly damaging the piezoelectric materials on the upper and lower sides of the base beam. This phenomenon was significant in the thicker piezoelectric beams. An increase in thickness rendered the piezoelectric beams more susceptible to the influence of non-linear effects during vibration [33]. It is hypothesized that the thinner piezoelectric beams would be more likely to maintain a more stable vibration mode during vibration. The thinner piezoelectric beams exhibited reduced inertia and augmented flexibility during vibration, enabling superior adaptation to variations in external excitation.

#### 3.1.2. Resistance Correlation Analysis

**Figure 6:**
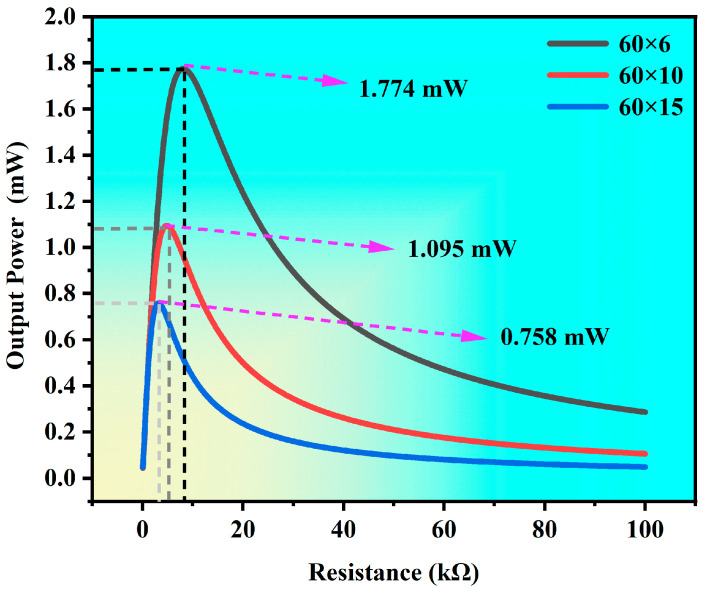
The correlation between the output power and the load of the single-wing piezoelectric beam near the first-order resonant frequency.

Figure 6 depicts the correlation between the output power and the load of the single-wing piezoelectric beam near the first-order resonant frequency. When the load resistance was relatively small, the output power increased with the resistance. However, the output power gradually decreased when the resistance increased to a certain level.

Output power is the product of voltage and current [34]. As the load resistance increased, the voltage increased, but the current decreased. The change in the output power depends on the relative rates of change between the two. This inflection point indicates the position corresponding to the maximum output power of the single-wing piezoelectric beam; the load resistance at this point is the optimum load resistance for the structure. Under their respective optimum load resistance conditions, the output power of the 6 mm-wide single-wing piezoelectric beam was 1.016 mW higher than that of the 15 mm-wide beam.

#### 3.1.3. Frequency Correlation Analysis

**Figure 7:**
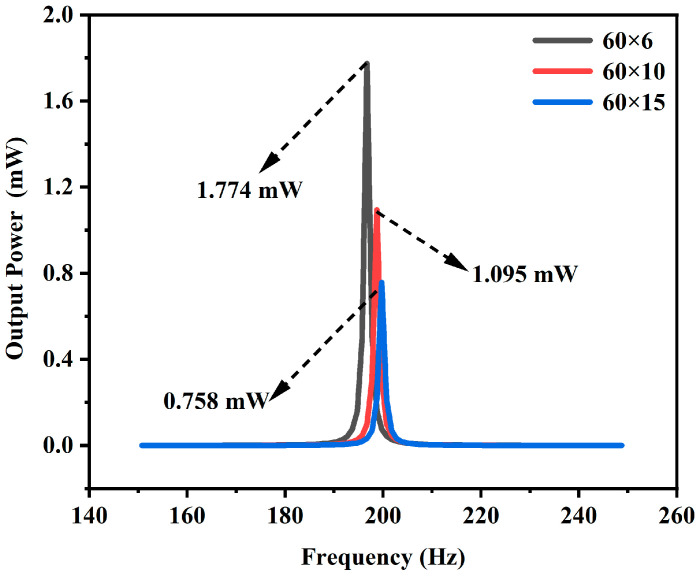
The correlation between the output power and the frequency of the single-wing piezoelectric beam near the first-order resonant frequency.

Figure 7 depicts the correlation between the output power and the frequency of the single-wing piezoelectric beam near the first-order resonant frequency. The output power showed an upward trend with increased vibration frequency until reaching a peak point, i.e., the optimal operating frequency [35]. At this frequency, the piezoelectric beam can maximize the conversion of mechanical into electrical energy, achieving maximum energy conversion efficiency.

When the base beam material was beryllium bronze, the optimal operating frequencies resulted in optimal output powers of 1.774 mW, 1.095 mW, and 0.758 mW for widths of 6 mm, 10 mm, and 15 mm, respectively. When the width increased by 233%, the optimal output power decreased by 57.27%. With the single-wing piezoelectric beam width of 6 mm, the average power could reach 0.465 mW within the bandwidth of 194–200 Hz. The output power decreased as the excitation frequency increased beyond this peak. An excessively high excitation frequency caused the vibration modes inside the piezoelectric beam to become more complex, affecting the effective energy conversion. As the width increased, the output power at the peak point gradually decreased. When the width of the piezoelectric beam increased by 150%, the output power decreased by 57.27%. This phenomenon, i.e., that an increase in the width of the piezoelectric beam decreases its output power, also corroborates the results in Section 3.1.2.

#### 3.1.4. Load Correlation Analysis

**Figure 8:**
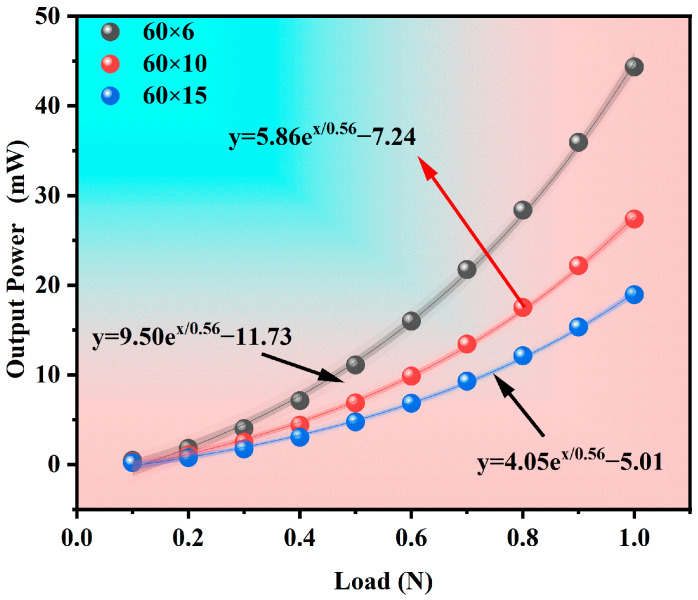
The correlation between the output power and the applied load of the single-wing piezoelectric beam near the first-order resonant frequency.

Figure 8 depicts the correlation between the output power and the applied load of the single-wing piezoelectric beam near the first-order resonant frequency. As the load increased, the output power showed an exponential growth trend. When the external load was 0.2 N, the output power of the beryllium-bronze piezoelectric beam with a width of 6 mm was 1.774 mW, corresponding to the results in Section 3.1.2 and Section 3.1.3. The difference in output power between the 6 mm-wide beam and the other two beams gradually increased with load.

When an external force is applied to a piezoelectric material, the internal charges are redistributed, generating voltage and current due to the piezoelectric effect [36]. In practical applications, the effective utilization of power is impeded by various factors. These include the material’s internal resistance, the external circuit’s connection method, and various losses during energy conversion. This portion of the power is designated as wasted power. As the load increases, the stress-strain state within the piezoelectric material undergoes significant alterations. Consequently, the charge distribution becomes more intricate, causing additional substantial fluctuations in current and voltage. These fluctuations augment the non-linear effects during energy conversion and aggravate the external circuit’s internal losses and matching issues. As a result, the wasted power grows exponentially.

The discrepancy in output power among piezoelectric beams of varying widths exhibited an incremental rise as the load magnitude increased. From a physical perspective, the energy conversion efficiency of piezoelectric materials is closely related to their size, shape, and stress state [37]. As the load increases, the discrepancy in output power between the piezoelectric beams with reduced widths and those with augmented widths gradually amplifies.

Equations (4)–(6) represent the fitting formulas for the output power of beryllium-bronze piezoelectric beams with widths of 6 mm, 10 mm, and 15 mm, respectively, as a load function. The gradient and the y-intercept of the formula increase accordingly with the width of the single-wing piezoelectric beam.

(4) y=9.50ex0.56−11.23

(5) y=5.86ex0.56−7.24

(6) y=4.05ex0.56−5.01

#### 3.1.5. Experimental-Simulation Correlation Analysis

**Figure 9:**
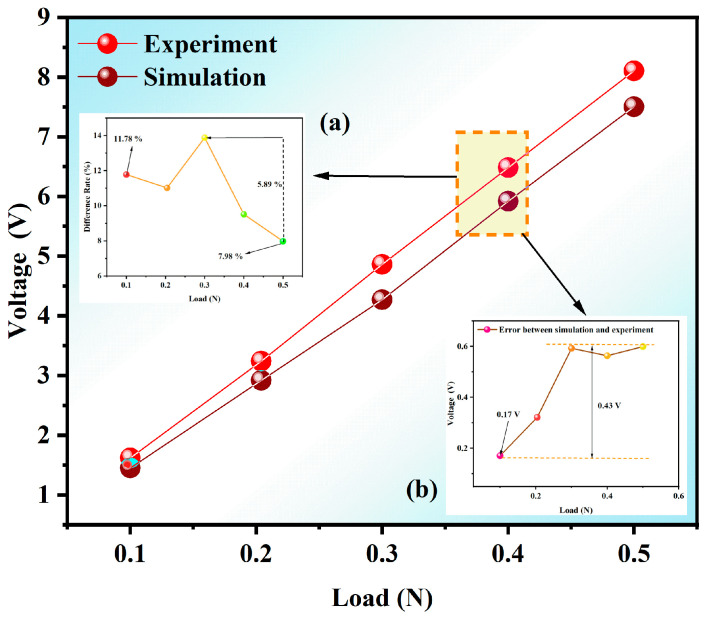
The 60 × 6 comparison chart of the experimental and simulation results for the single-wing piezoelectric beam. (a) Difference rate; (b) error between simulation and experiment.

Figure 9 shows a comparison chart of the experimental and simulation results for the 60 × 6 type of single-wing piezoelectric beam. According to Figure 9, the experimentally measured voltage of the single-wing piezoelectric beam agrees with the simulation results regarding the variation trend—both voltages increase with load. However, the experimental voltage is always lower than the simulation value, indicating that the simulation model may overestimate the actual performance or energy loss in the experiment. As demonstrated in Figure 9a, the difference rate first increases and then decreases between the experimental and simulated outcomes with an increase in load. Figure 9b clarifies a two-stage discrepancy between experimental and simulated results. At 0.1–0.2 N, there is a gradual escalation in the discrepancy. At 0.2–0.3 N, there is a gradual escalation in the discrepancy, but the slope of the difference curve tends to increase. Reaching a peak value of 0.59 V at 0.3 N. At 0.3–0.5 N, the difference tends to stabilize. However, there is a sudden change in the difference between the experimental and simulated results at 0.3 N. This phenomenon is especially evident in the non-linear response part in the medium load interval. Simultaneously, the energy loss in the experiment (such as mechanical friction or material defects) also needs to be more accurately controlled.

### 3.2. Magnetic-Coupling Performance

#### 3.2.1. Theoretical Model

**Figure 10:**
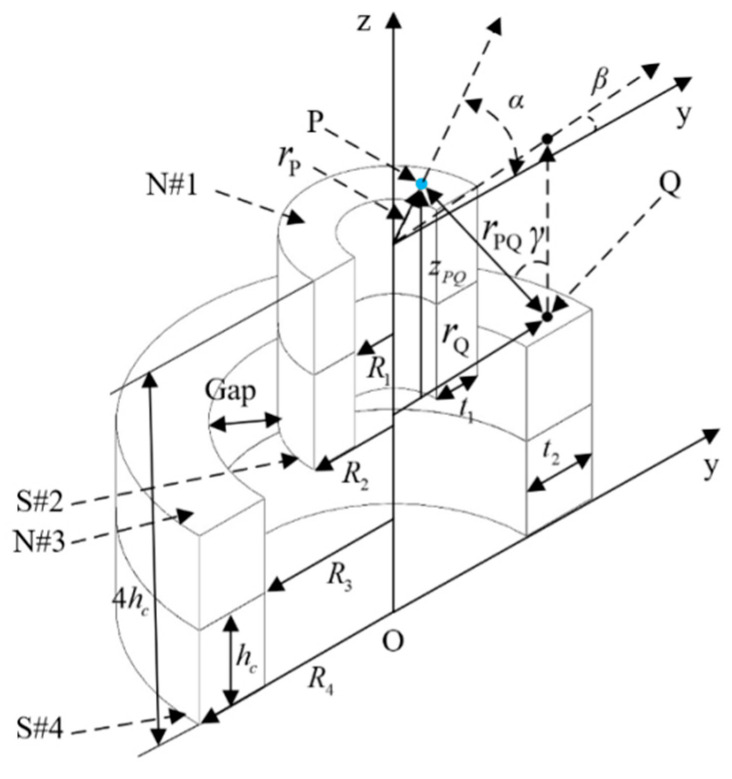
Force analysis diagram of the cross-section of the magnetic-coupling part.

The magnetic coupling structure is shown in Figure 10. The axes of the upper and lower magnetic rings are coaxial, i.e., the z-axis and both magnetization directions are oriented along the positive z-axis. It is assumed that the lower magnetic ring of this mechanism is fixed, and the motion range of the upper magnetic ring is 0–2*h*_c_. The mutually repelling magnetic rings act as an axial bearing with negative stiffnesses.

In Figure 10, N represents the north pole of the magnetic ring; S represents the south pole of the magnetic ring; *R*_1_ is the inner radius of the upper magnetic ring; *R*_2_ is the outer radius of the upper magnetic ring; *R*_3_ is the inner radius of the lower magnetic ring; *R*_4_ is the outer radius of the lower magnetic ring; *h*_c_ is the height of a single magnetic pole; 4*h*_c_ is the height of the magnetic coupling structure; *t*_1_ is the thickness of the upper magnetic ring; *t*_2_ is the thickness of the lower magnetic ring; and *l_dr_* is the gap between the magnetic rings. The gap is the difference between the outer radius of the lower ring and the inner radius of the upper ring, as shown in Equation (7):

(7) $$l_{dr}=R_{3}-R_{2}$$

According to the equivalent magnetic charge method [38], the upper and lower surfaces of the magnetic ring are represented by charged surfaces. Parts #1 and #3 are taken as examples. The magnetic charge Q_P_ at any point P on the #1 surface and the magnetic charge Q_Q_ at any point Q on the #3 surface can be expressed as follows:

(8) $$Q_{P}=\rho_{P}r_{P}d\alpha dr_{P}$$

(9) $$Q_{Q}=\rho_{Q}r_{Q}d\beta dr_{Q}$$

where *r*_p_ and α are the polar coordinates of the point P on surface #1; *r*_Q_ and *β* are the polar coordinates of the point Q on surface #3; *ρ*_Q_ is the surface density of magnetic charge on surface #1; and *ρ*_P_ is the surface density of magnetic charge on surface #3.

According to the electromagnetic theory [39], the magnetic field intensity Q_P_ generated by the magnetic charge Q at point ***E***_P_(*t*) when the time is *t* is given by Equation (4):

(10) $$E_{P}(t)=\frac{Q_{P}r_{PQ}}{4\pi \mu_{0}r_{PQ}^{3}(t)},$$

where ***r***_PQ_ (*t*) is the vector from point P to point Q; *μ*_0_ is the vacuum permeability (*μ*_0_ = 1); and *r*_PQ_ (*t*) is the distance from point P to point Q.

According to Equation (10) and the electromagnetic theory [39], the surface density *ρ* of the permanent magnet’s magnetic charge is equal to the residual magnetic induction intensity. The axial component *F*_PQz_(*t*) of the magnetic force *F*_PQ_(*t*) between points P and Q, represented by the residual magnetic induction intensity, is given by Equation (11):

(11) $$dF_{\mathrm{PQz}}(t)=\frac{B_{\mathrm{rP}}B_{\mathrm{rQ}}r_{P}r_{Q}d\alpha d\beta dr_{P}dr_{Q}}{4\pi \mu_{0}r_{\mathrm{PQ}}^{3}(t)}z_{\mathrm{PQ}}(t),$$

where z_PQ_(*t*) is the component of the distance *r*_PQ_(*t*) from point P to point Q along the z-axis, and **z** is the unit vector in the z-axis, $z=\left[\begin{array}{ccc} 0 & 0 & 1 \end{array}\right]$:

(12) $$dF_{24z}(t)=dF_{\mathrm{PQz}}(t)$$

Similarly, the axial components of the magnetic force on #2 and #3 surfaces and #1 and #4 surfaces are d*F*_23z_(*t*) and d*F*_14z_(*t*), respectively; the components of the spacing between #2 and #3 surfaces and #1 and #4 surfaces in the z-axis direction are *r*_23z_ and *r*_14z_, respectively; the components of the spacing between #2 and #4 surfaces and #1 and #3 surfaces in the z-axis direction are *r*_24z_ and *r*_13z_, respectively. Therefore, for surfaces #1, #2, #3, and #4, the axial magnetic force d*F_ij_*_z_(*t*) between any two surfaces and the z-axis direction components of the spacing between any two surfaces are *r_ij_*_z_. Parameter *i* is used to denote surfaces #1 and #2, and *j* is used to denote surfaces #3 and #4.

(13) $$dF_{ijz}(t)=\frac{B_{ri}B_{rj}r_{i}r_{j}d\alpha d\beta dr_{i}dr_{j}}{4\pi \mu_{0}r_{ij}^{3}(t)}z_{ij}(t),\;i=1,2;\;j=3,4,$$

(14) $$r_{ij}(t)=\left|r_{ij}(t)\right|=\sqrt{z_{ij}^{2}(t)+{\left(r_{i}\sin\alpha -r_{j}\sin\beta \right)}^{2}+{\left(r_{i}\cos\alpha -r_{j}\cos\beta \right)}^{2}},\;i=1,2;\;j\;=3,4,$$

(15) $$z_{23}(t)=2h_{c}-z_{\mathrm{PQ}}(t),$$

(16) $$z_{14}(t)=2h_{c}+z_{\mathrm{PQ}}(t),$$

(17) $$z_{13}(t)=z_{\mathrm{PQ}}(t).$$

In the discussion of this paper, the outer ring is set as a fixed constraint condition. In contrast, the inner ring undergoes dynamic displacement along the z-axis direction. When the inner ring deviates from its static equilibrium position, O, by a certain distance, z, its force condition changes significantly. Specifically, in the positive z-axis direction, the inner ring is affected by the combined action of three magnetic forces: d*F*_13z_(*t*), d*F*_23z_(*t*), and d*F*_24z_(*t*). These forces are all characterized by a positive thrust effect on the inner ring. Conversely, in the negative z-axis direction, the inner ring is affected by an attractive force, which attempts to cause the inner ring to displace in the negative direction d*F*_14z_(*t*).

According to the above force analysis, the principle of vector synthesis must be adopted. The components of these four forces in the z-axis direction must be accumulated to accurately solve the resultant magnetic force d*F*_z_(*t*) of the inner ring in the z-axis direction. Therefore, the differential expression of the resultant magnetic force d*F*_z_(*t*) can be defined as the sum of the components of these four forces in the z-axis direction. The integral is performed to obtain Equation (18). This expression provides an important mathematical tool for in-depth study of the dynamic behavior and force characteristics of the inner ring:

(18) $$F_{z}(t)=\varphi_{1}\sum_{i=1}^{2}\sum_{j=3}^{4}\int_{0}^{2\pi }\int_{0}^{2\pi }\int_{R_{1}}^{R_{2}}\int_{R_{3}}^{R_{4}}\left(\frac{B_{ri}B_{rj}r_{i}r_{j}z_{ij}}{4\pi \mu_{0}r_{ij}^{3}(t)}\right)d\alpha d\beta dr_{i}dr_{j},$$

where $\varphi_{1}$ is the size effect coefficient.

The restoring force of the magnetic coupling module is obtained based on Newton’s third law as Equation (19):

(19) $$F_{z}(t)=-F_{f}(t)$$

The stiffness characteristics of the magnetic coupling module can be revealed and derived by conducting an in-depth exploration of the differential relationship between the magnetic force *F*_f_(*t*) acting on the inner ring in the z-axis direction and its displacement z. In other words, Equation (20) can be obtained [40]. This expression can accurately describe the stiffness variation of the magnetic coupling module under different displacements:

(20) $$k_{c}=\frac{dF_{f}(t)}{dz}=-\frac{dF_{z}(t)}{dz}$$

#### 3.2.2. Effect of Remanence Intensity

**Figure 11:**
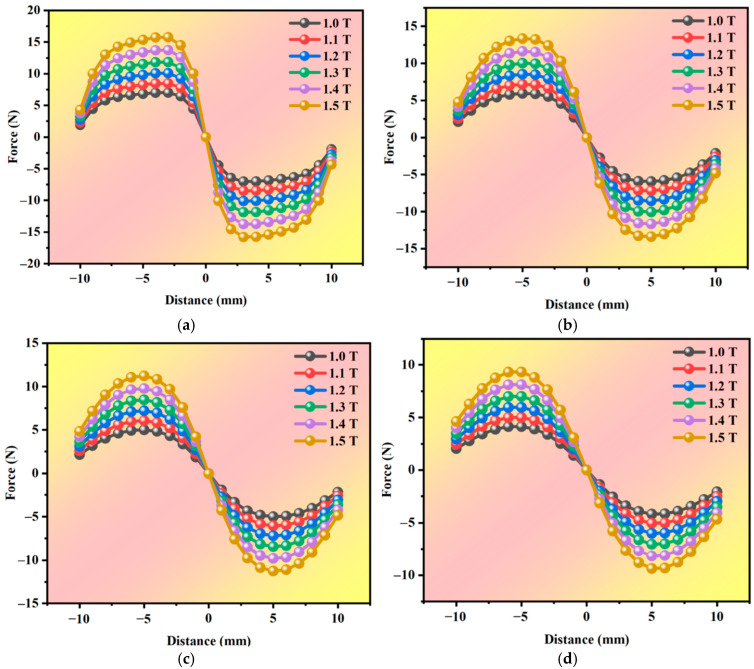

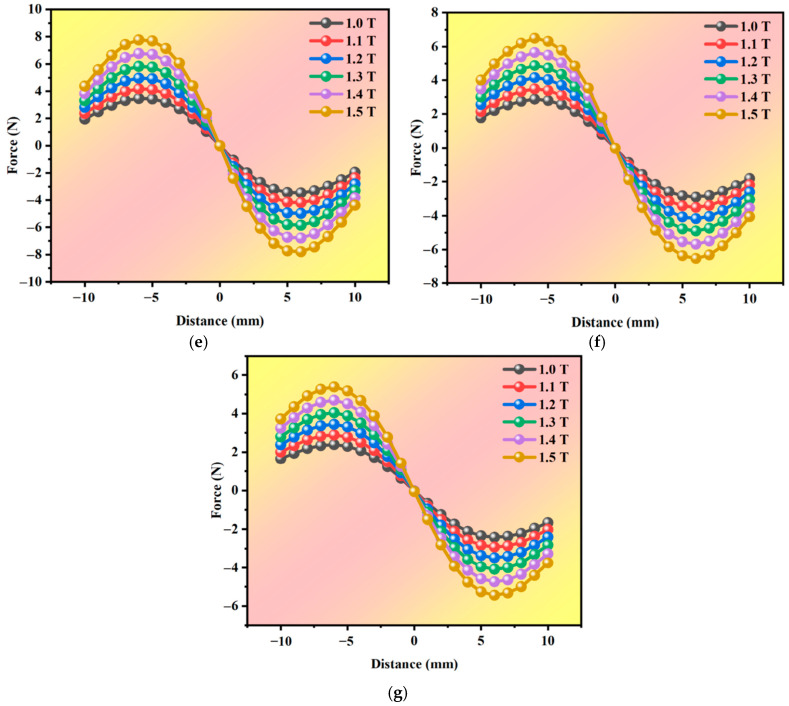
The influence of remanence intensity on the restoring force of the magnetic ring at different magnetic gaps. (**a**) Magnetic gap of 1 mm, (**b**) magnetic gap of 2 mm, (**c**) magnetic gap of 3 mm, (**d**) magnetic gap of 4 mm, (**e**) magnetic gap of 5 mm, (**f**) magnetic gap of 6 mm, and (**g**) magnetic gap of 7 mm.

Figure 11 represents the influence of remanence intensity on the restoring force of the magnetic ring at different magnetic gaps. As illustrated in Figure 11a, motion initiation in the positive z-axis direction by the upper magnetic ring caused a concomitant alteration in the relative positions between the magnetic rings, an augmentation in the restoring force of the magnetic ring, and a corresponding increase in the restoring force of the magnetic ring. After attaining its peak value, the restoring force gradually decreased. In the event of the upper surfaces of the two magnetic rings attaining a state of parallelism, the restoring force became zero. It should be noted that the restoring force between the magnetic rings underwent a reversal in direction when passing the critical position. Concurrently, the restoring force commences an upward trend. As the displacement increased, the distance between the upper and lower magnetic rings initially decreased. Furthermore, the restoring force of the magnetic rings exhibited an initial upward trend followed by a subsequent decrease. Lastly, it should be noted that the restoring force of the magnetic rings exhibited symmetry with respect to 0 mm.

In the initial phase, the restoring force of the magnetic ring increased with the remanence intensity. Increasing the remanence intensity caused a change in the initial magnetic force between the magnetic rings, decreasing the restoring force [41]. The direction of the restoring force reversed at a distance of 0 mm from the magnetic ring, entering the second stage. This phenomenon can be attributed to the enhancement of magnetic force between the magnetic rings, resulting from increased remanence intensity [42]. The variation trend of the magnetic restoring force in Figure 11b exhibited a congruent pattern with that of the magnetic ring restoring force in Figure 11a. This finding substantiates the close relationship between the magnetic ring restoring force and the magnetic force. The change in the restoring force was also influenced by the remanence intensity, exhibiting a two-stage variation trend analogous to the restoring force of the magnetic ring.

As demonstrated in Figure 11c–g, the restoring force of the magnetic ring is directly proportional to the remanence intensity when the magnetic gap condition is met. In a manner analogous to Figure 11a,b, the phenomenon could be subdivided into two distinct stages. This trend is consistent with the trends observed in Figure 11a,b, verifying the similarity between the restoring force of the magnetic ring with different magnetic gaps and the remanence intensity or the displacement of the upper magnetic ring [42].

**Figure 12:**
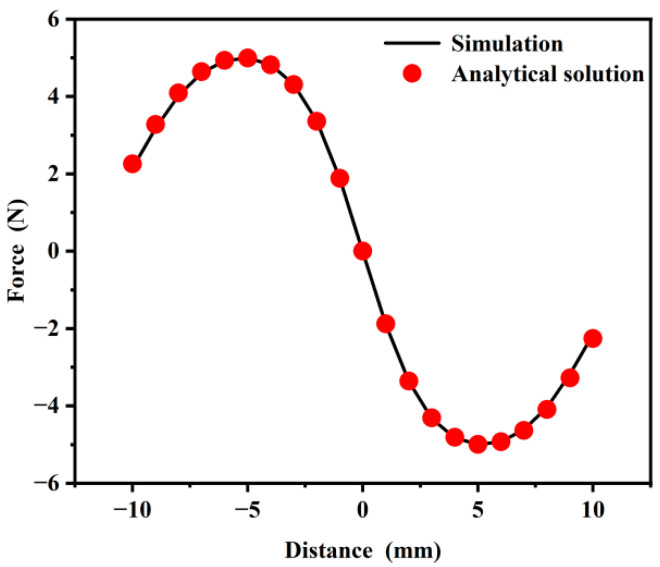
Analytical solution and finite element result curves of the type 5–8 magnetically coupled module with a remanence intensity of 1.0 T.

Figure 12 shows an analytical solution and finite element result curves of the Type 5–8 magnetically coupled module with a remanence intensity of 1.0 T. As shown in Figure 12, the theoretical prediction of the magnetic force in the magnetic coupling structure, derived using the equivalent magnetic charge method, agrees with the simulation results, indicating the high accuracy of this theoretical prediction.

**Figure 13:**
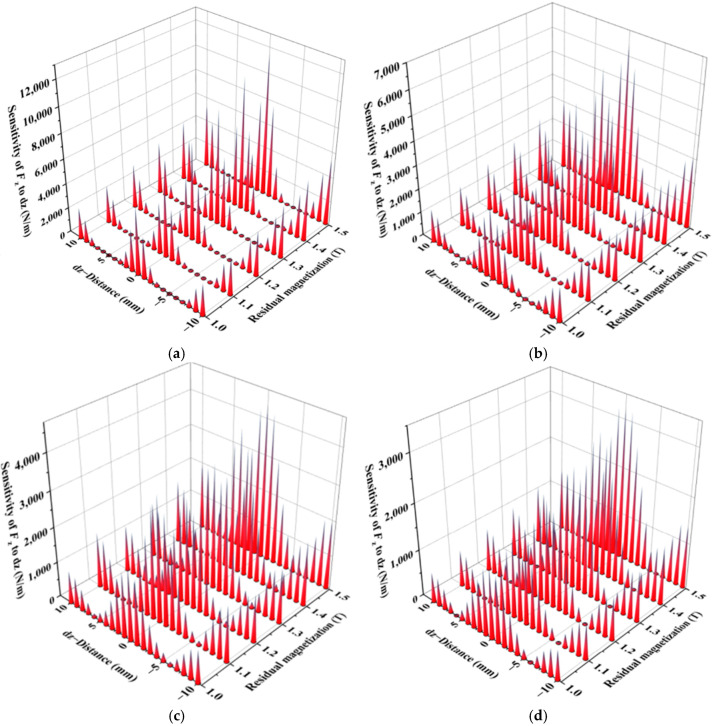

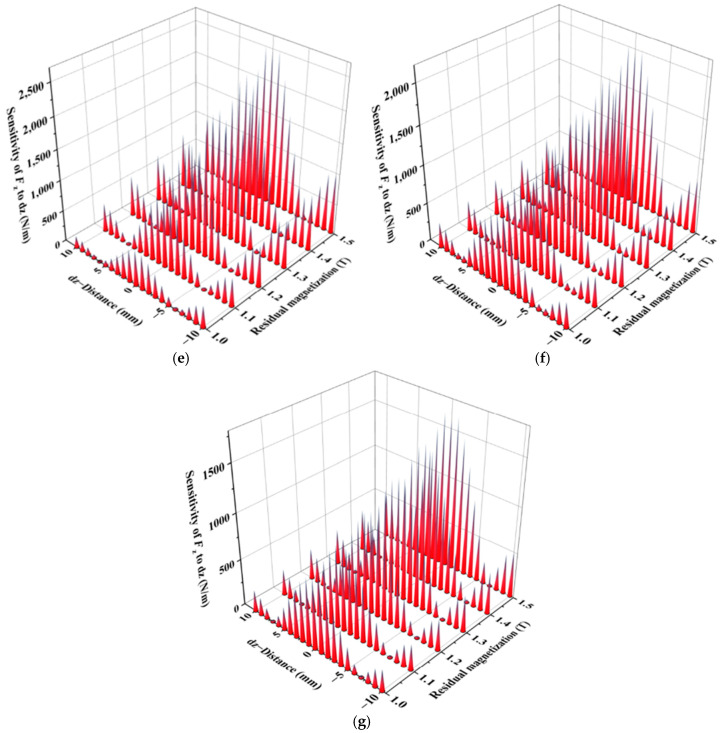
Sensitivity of *F*_z_ relative to dz at different magnetic gaps. (**a**) Magnetic gap of 1 mm, (**b**) magnetic gap of 2 mm, (**c**) magnetic gap of 3 mm, (**d**) magnetic gap of 4 mm, (**e**) magnetic gap of 5 mm, (**f**) magnetic gap of 6 mm, and (**g**) magnetic gap of 7 mm.

Figure 13 shows the sensitivity of *F*_z_ relative to dz at different magnetic gaps. Parameter *F*_z_ represents the restoring force of the magnetic ring, and dz represents the moving distance of the upper magnetic ring. As demonstrated in Figure 13, the sensitivity of the field strength, denoted as *F*_z_, with respect to the displacement, represented by dz, exhibited an initial decrease followed by an increase as the displacement varied for varying remanence intensities. This sensitivity was symmetrical about the zero point.

When the displacement ranged from 4 mm to 6 mm, the response of the former to the latter became relatively insensitive. This response indicates that a minor alteration in displacement did not substantially change the system’s response. When the displacement ranged from −2 to 2 mm and 8 to 10 mm, the response of the former to the latter became sensitive. This sensitivity was particularly pronounced within the −2–2 mm displacement range, where a minor alteration in displacement could result in a substantial change in response.

Conversely, when the displacement was held constant, an enhancement in the remanence intensity increased the sensitivity of the force with respect to the displacement. The enhancement of the remanence intensity rendered the response of the restoring force to the displacement more sensitive. According to physics principles, the remanence intensity determines the magnetic ring’s initial magnetization state or mechanical state within the magnetic force system [41]. As the displacement changes, the magnetization or mechanical state of the magnetic ring will change accordingly, changing the remanence. An increase in remanence intensity means that the initial state of the magnetic ring is more sensitive to external conditions. If the displacement changes, the change in the restoring force is significant.

#### 3.2.3. Effect of the Magnetic Gap

**Figure 14:**
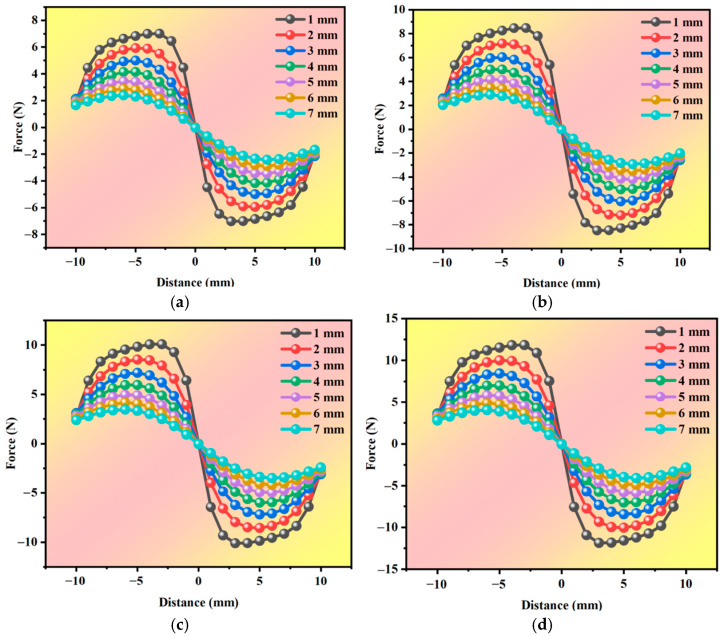

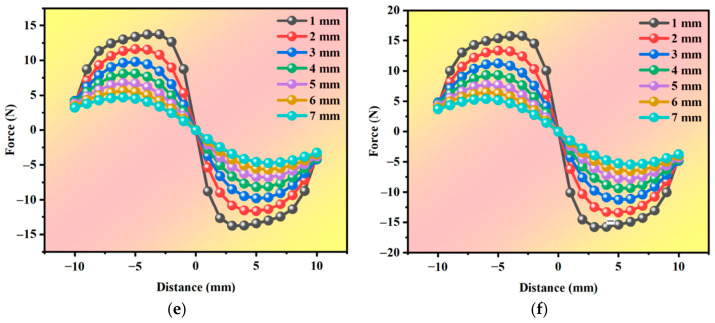
The influence of magnetic gap on the restoring force at different remanence intensities. (**a**) Remanence intensity of 1.0 T, (**b**) remanence intensity of 1.1 T, (**c**) remanence intensity of 1.2 T, (**d**) remanence intensity of 1.3 T, (**e**) remanence intensity of 1.4 T, and (**f**) remanence intensity of 1.5 T.

Figure 14 depicts the influence of the magnetic gap on the restoring force at different remanence intensities. As shown in Figure 14a, the restoring force of the magnetic ring changes with an increase in the magnetic gap; its variation trend could be divided into two stages. In the first stage, the restoring force of the magnetic ring showed a gradually increasing trend with an increase in the magnetic gap. The magnetic force between the magnetic rings was relatively strong when the magnetic gap was small. Therefore, the resulting restoring force was large. As the magnetic gap increased, the magnetic force between the rings gradually weakened, decreasing the restoring force. When the magnetic gap was 6 mm, the difference in restoring force between the magnetic rings with a 6 mm magnetic gap and those with a 7 mm magnetic gap was only 8.53%. At the initial distance, i.e., when the distance was 10 mm, the magnetic forces between the magnetic rings with different magnetic gaps were similar.

As the distance from the lower magnetic ring decreased, the difference in magnetic forces between the magnetic rings with different magnetic gaps gradually increased. When the distance was 5 mm, the difference in magnetic forces between the magnetic rings with different magnetic gaps reached the maximum value. Simultaneously, the magnetic forces between the magnetic rings with different magnetic gaps reached their maximum values. The difference in restoring force between the magnetic rings with different magnetic gaps gradually decreased with the distance. When the distance between the magnetic rings was 0 mm, the direction of the restoring force was reversed, and the restoring force entered the second stage. At this stage, the variation pattern of the magnetic gap was similar to that of the magnetic ring restoring force.

According to Figure 14b–f, the variation trend in the magnetic force under different remanence intensities is the same as that of the restoring force of the magnetic ring in Figure 14a. When the magnetic gap increased, the restoring force increased and then decreased. Once the direction of the restoring force was reversed, the restoring force first increased and then decreased.

**Figure 15:**
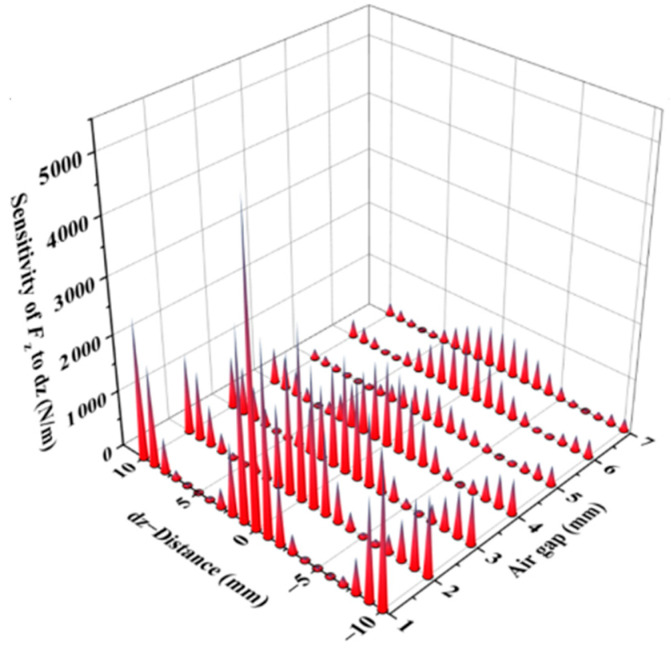
Remanence intensity of 1.0 T and the sensitivity of *F*_z_ relative to dz at different air gap.

The sensitivity of *F*_z_ relative to dz at different air gaps and the remanence intensity of 1.0 T is shown in Figure 15. The sensitivity of *F*_z_ to dz is symmetrical about the zero point, first decreasing and then increasing with displacement. A small change in displacement could cause a large change in *F*_z_ in the range of −2 mm to 2 mm. When dz was the same, the sensitivity of *F*_z_ with respect to dz also showed an increasing trend with the magnetic gap. As the magnetic gap increased, the sensitivity of the restoring force to displacement decreased. An increase in the magnetic gap meant that the initial state of the magnetic ring was more difficult to influence via external conditions. It has been demonstrated that, as the displacement increased, the restoring force of the ring with a large magnetic gap increased less considerably than that of the ring with a small magnetic gap.

#### 3.2.4. Effect of Stiffness

Due to the structural constraints imposed by spatial dimensions, the energy-harvesting structure dominated by positive stiffness has a relatively low degree of match between its high natural frequency and the environmental vibration frequency. Incorporating a negative-stiffness structure into the system can lower its natural frequency. This effect allows it to match the low-frequency vibration environment and enhance the energy-harvesting efficiency. Let the stiffness be $k_{+}$ and the negative stiffness be $k_{-}$. If $\left|k_{-}\right|$ < $\left|k_{+}\right|$; the equivalent stiffness of the system will be significantly reduced, and the natural frequency of the system will be reduced to a relatively low value. If $k_{-}$ > $\left|k_{+}\right|$, the system may experience a divergence situation. In other words, the mass block will continue to move away from the equilibrium position after a small disturbance, causing the system to malfunction. If the absolute value of the negative stiffness gradually increased and approached the positive stiffness (while remaining below the level of the positive stiffness), this structure could release the negative stiffness of the magnetic ring relatively optimally. However, excessive negative stiffness could negatively affect the structure and cause structural failure [40].

If the value of negative stiffness is too high, the structure will have difficulty maintaining its stable geometric shape and load-carrying capacity. This phenomenon can lead to excessive displacement, deformation, or even structure failure. If the absolute value of the negative stiffness is close to but less than the positive stiffness, the negative stiffness can reduce the overall stiffness of the structure. In the structural design of this study, negative stiffness is introduced to reduce the overall stiffness, lower the natural frequency of the system, and expand the frequency range of the optimal output characteristics. These parameters allow the structure to better adapt to external excitation and optimize its dynamic performance. Simulation controls the magnitude of negative stiffness precisely to ensure its absolute value is close to but lower than positive stiffness. This approach can avoid the risk of structural failure caused by excessive negative stiffness and utilize the characteristics of negative stiffness to control structural stiffness, meeting the diverse requirements of practical engineering.

According to [43], the positive stiffness of the structure is taken as the force that causes a unit displacement at the free end of a single-wing piezoelectric beam:

(21) $$F=\frac{3EI\Delta }{l^{3}},$$

where Δ is a unit displacement.

**Figure 16:**
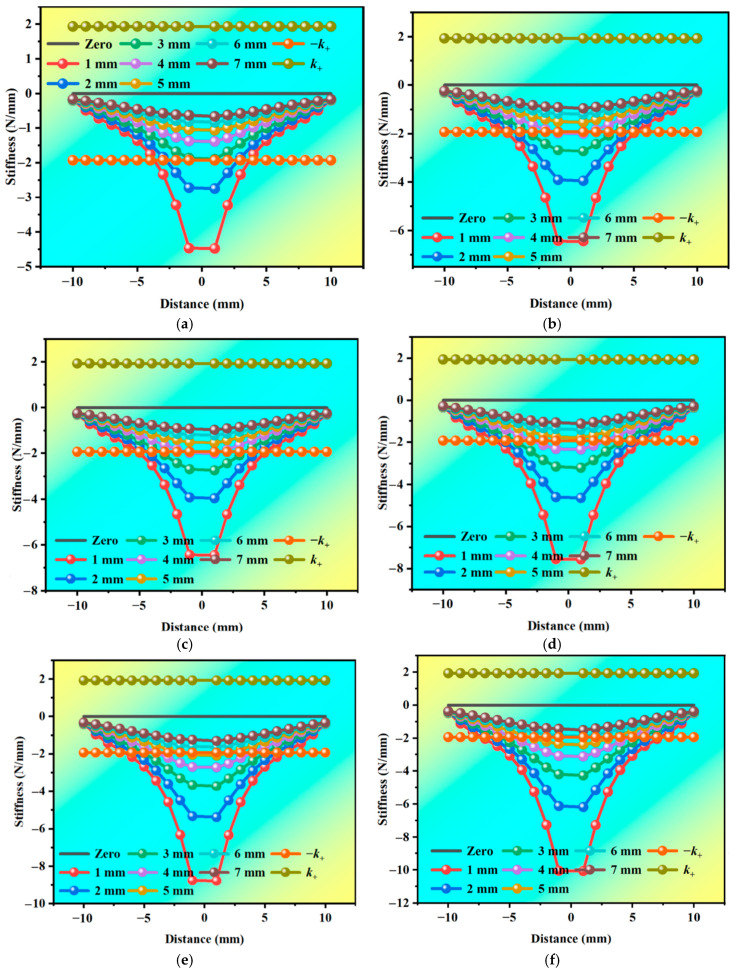
The impact of the gap on the stiffness of magnetically coupled structures at different remanence intensities. (**a**) Remanence intensity of 1.0 T, (**b**) remanence intensity of 1.1 T, (**c**) remanence intensity of 1.2 T, (**d**) remanence intensity of 1.3 T, (**e**) remanence intensity of 1.4 T, and (**f**) remanence intensity of 1.5 T.

Figure 16 shows the impact of the gap on the stiffness of magnetically coupled structures at different remanence intensities. According to Figure 16a, the absolute value of the negative rigidity of the magnetic rings gradually decreased with a gradual increase in the distance between the magnetic rings. Furthermore, the variation law of the negative rigidity of the magnetic rings with respect to the distance was symmetrical about the zero point. In other words, as the distance changes, the variation trend of the absolute value of the negative rigidity of the magnetic rings is mirror symmetrical.

As the magnetic gap decreases and the distance gradually increases, the absolute value of the negative rigidity of the magnetic rings gradually increases. As the magnetic gap decreases, the interaction area between the magnetic rings becomes compact, and the interaction of the magnetic fields becomes more intense, increasing the absolute value of the negative stiffness. The increase in remanence intensity increases the magnetism of the magnetic rings, increasing the negative stiffness effect during their interaction. The absolute value of the negative stiffness of the magnetic rings increases with a decrease in the magnetic gap and an increase in the distance-related factor.

When the remanence intensity was 1.0 T, and the magnetic gap was 3 mm, the maximum absolute value of the negative stiffness of the magnetic coupling was only 0.03 N/mm less than the positive stiffness of the structure. When the magnet-ring gap was less than 3 mm (specifically, when the gap was 1 mm and 2 mm), the maximum absolute values of the negative stiffness of the magnetic coupling were 131.44% and 40.85% higher than the positive stiffness, respectively. When the gap between the magnetic rings was greater than 3 mm (specifically, when the gap was 4–7 mm), the maximum absolute values of the negative stiffness of the magnetic coupling were 28.05%, 44.85%, 56.81%, and 65.39% lower than the positive stiffness for gaps of 4 mm, 5 mm, 6 mm, and 7 mm, respectively.

According to Figure 16b–f, the variation laws of the absolute value of the negative stiffness of the magnetic rings with respect to the magnetic gap and remnant magnetic intensity are similar. When the gap is close to the value corresponding to the positive stiffness value, the absolute value of the negative stiffness is greater than the positive stiffness. Therefore, the magnetic coupling module with a remanence intensity of 1.0 T and a magnetic gap of 3 mm was selected for the negative stiffness module of the energy harvester. On one hand, this selection fully exploits the performance of the magnetic coupling module. On the other hand, it avoids the structure being subjected to excessive loads and producing excessive displacements. In this instance, the absolute value of the negative stiffness would become greater than the positive stiffness, leading to the failure of the structure.

## 4. Conclusions

This paper established a single-wing piezoelectric beam model and a finite element model of the magnetic coupling module. The modal and output characteristics of the single-wing piezoelectric beam and the stress characteristics of the magnetic-coupling negative module were analyzed. As the length of the single-wing piezoelectric beam increases, the contribution of its width to the modal frequency gradually decreases. The width has a relatively small effect on the low-order frequencies and a relatively large effect on high-order frequencies. Thickness significantly affects the eigenfrequency. The load is exponentially related to the output power.

As the magnetic gap increases, the peak value of the magnetic field intensity, which corresponds to the negative stiffness of the magnetic coupling, gradually decreases. An increase in remanence intensity means that external conditions more easily influence the initial state of the magnetic ring. The variation in the axial magnetic force becomes more pronounced with a change in the displacement. For different magnetic gaps, the sensitivity of *F*_z_ with respect to dz first decreases to a minimum value and then increases with displacement. As the magnetic gap decreases and the displacement gradually increases, the absolute value of the negative stiffness of the magnetic ring gradually increases. A reduction in the magnetic gap indicates that the interaction between the magnetic rings becomes compact. In contrast, the interaction of the magnetic fields becomes more intense, increasing the absolute value of the negative stiffness. This study focuses on the modal and output properties of piezoelectric modules, as well as the performance of magnetically linked modules. In future work, the piezoelectric and magnetic linkage modules will be integrated to establish theoretical and experimental models of the magnetic linkage biplane negative stiffness collector. The assembly and experimental testing of the magnetically linked biplane negative stiffness collector will then follow.

## Figures and Tables

**Table 1:** Parameter scheme for the simulation model.

Variable		Scope
Load (N)		0.1–1/step 0.1
Size of piezoelectric sheet	Length (mm)	40–120/step 10
	Width (mm)	5–20/step 1
	Thickness (mm)	0.2
Single-wing piezoelectric beam	Thickness (mm)	0.5–2/step 0.5
Piezoelectric sheet material		Lead Zirconate Titanate Piezoelectric (PZT-5H)
Magnetic ring material		N52
Magnetic ring gap (mm)		1–7/step 1
Residual magnetism intensity (T)		1.0–1.5/step 0.1
Magnetic ring thickness (mm)		2
Magnetic ring height (mm)		10
Single-wing piezoelectric beam		Beryllium bronze

**Table 2:** Experimental parameters.

Single-Wing Piezoelectric Beam Model	Input Frequency (Hz)	Load (N)
60 × 6	73	0.1–1/step 0.1

**Table 3:** The single-wing piezoelectric beam mode.

Vibration Mode	Type 40	Type 50	Type 60
First	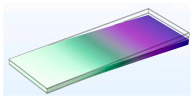	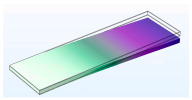	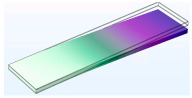
Second	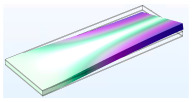	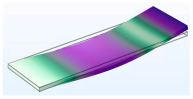	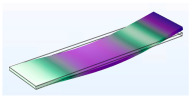
Third	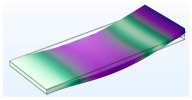	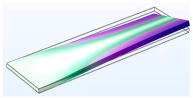	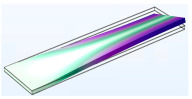
Fourth	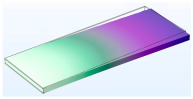	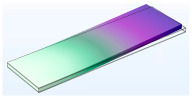	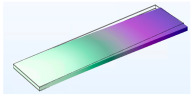

**Table 4:** The single-wing piezoelectric beam mode.

Vibration Mode	Type 6	Type 10	Type 15
First	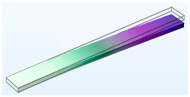	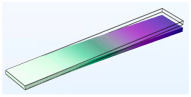	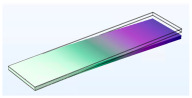
Second	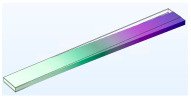	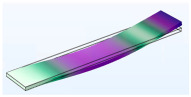	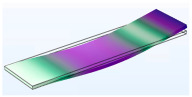
Third	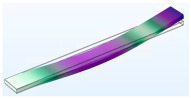	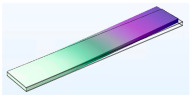	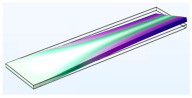
Fourth	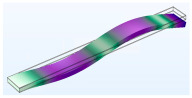	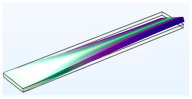	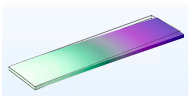

**Table 5:** The single-wing piezoelectric beam mode.

Vibration Mode	Type 0.5	Type 1	Type 1.5
First	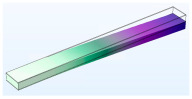	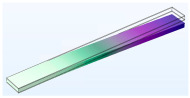	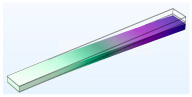
Second	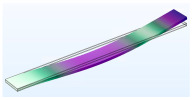	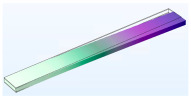	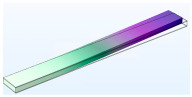
Third	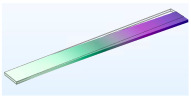	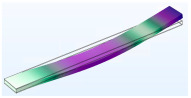	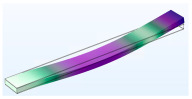
Fourth	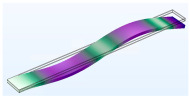	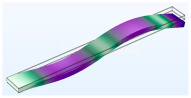	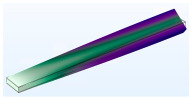

## Data Availability

The original contributions presented in this study are included in the article. Further inquiries can be directed to the corresponding author.
